# The Effect of Fungicide Protection on Mycotoxin Contamination and Microscopic Fungi in Spring Barley Grain Cultivated in Northeastern Poland

**DOI:** 10.3390/toxins18040164

**Published:** 2026-03-30

**Authors:** Agnieszka Pszczółkowska, Elżbieta Suchowilska, Michael Sulyok, Wolfgang Kandler, Adam Okorski, Rudolf Krska, Marian Wiwart

**Affiliations:** 1 Department of Genetics and Plant Pathophysiology, Faculty of Agriculture and Forestry, University of Warmia and Mazury in Olsztyn, Prawocheńskiego 17, 10-720 Olsztyn, Poland; agnieszka.pszczolkowska@uwm.edu.pl (A.P.); adam.okorski@uwm.edu.pl (A.O.); 2 Department of Plant Breeding and Bioresource Engineering, Faculty of Agriculture and Forestry, University of Warmia and Mazury in Olsztyn, pl. Łódzki 3, 10-724 Olsztyn, Poland; ela.suchowilska@uwm.edu.pl; 3 Institute of Bioanalytics and Agro-Metabolomics, Department of Agricultural Science (BOKU), BOKU University, Konrad Lorenz Str. 20, 3430 Tulln an der Donau, Austria; michael.sulyok@boku.ac.at (M.S.); wolfgang.kandler@boku.ac.at (W.K.); rudolf.krska@boku.ac.at (R.K.)

**Keywords:** fungicidal control, grain mycobiome, *Hordeum vulgare*, LC-MS/MS, mycotoxins

## Abstract

A three-year experiment was conducted over the years 2020–2022 to determine the spectrum of microscopic fungi colonizing the grain of two fungicide-treated cultivars of spring barley and the profiles of mycotoxins identified in grain. In comparison with the unprotected control, fungicide treatment significantly increased grain yield by an average of approximately 10% in cv. Atrika and approximately 20% in cv. Vermont. The most abundantly isolated species were *Alternaria alternata* and *Bipolaris sorokiniana*. Fungi of the genus Fusarium were also widely represented, accounting for 7% to 27% of all isolates, depending on the year. Each year, 45 secondary fungal metabolites produced mainly by Fusarium and Alternaria species were identified. Fungicide protection did not reduce the overall concentration of Fusarium toxins and even caused a slight increase, while contributing to a decrease in the levels of nivalenol-3-glucoside, nivalenol, and deoxynivalenol. Concurrently, the concentrations of group A trichothecenes and moniliformin increased. The grain of spring barley cv. Vermont contained higher levels of the major Fusarium toxins than the grain of cv. Atrika. Non-parametric Friedman ANOVA revealed significant differences between years for eight mycotoxin concentrations. These results confirm the complex effects of chemical protection on the composition of grain microflora and mycotoxin profiles, indicating the need for further research into interactions between cultivars, environmental conditions, and integrated plant protection strategies in the production of food and feed cereals to improve food safety.

## 1. Introduction

Barley (*Hordeum vulgare* L.) ranks fourth in global cereal production after wheat, rice, and maize. In 2023, a total of 146 million tons of barley grain were harvested from a global barley cultivation area of 46 million hectares, indicating an average yield of more than 3 tons per hectare (3.15 t/ha). The leading barley producers are the Russian Federation, France, and Germany [1]. In Poland, barley is cultivated on an area exceeding 647,000 ha, and total production reached 2.8 million tons in 2023, with an average yield of 4.4 t/ha [1]. Nearly half of the total barley cultivation area (295,000 ha, i.e., 45.6%) was sown with spring barley, from which a total of 1.1 million tons of grain were harvested at an average yield of 3.79 t/ha [2,3]. Barley is a versatile crop. Cultivars with high protein content and low starch content in grain are grown for animal feed and for food, while the grain of cultivars with high starch content and low protein content is malted and fermented in the production of beer and distilled alcoholic beverages; it is also used as seeds [3,4].

Fungal diseases, particularly those caused by fungi of the genus *Fusarium*, play a key role among the multiple factors influencing grain yield and quality in various cereal species [5,6,7,8,9,10]. Fusarium head blight (FHB), caused by numerous *Fusarium* species, is a devastating disease of small-grain cereals, including barley, resulting in substantial yield losses and grain contamination with mycotoxins that pose a global threat to food and feed safety [5,9,11,12]. In a study by Drakopoulos et al. [5], the dominant *Fusarium* species colonizing barley grain was *Fusarium graminearum*, followed by *F. avenaceum* and *F. poae*, whereas Henriksen and Elen [13] most frequently identified *F. avenaceum* and *F. tricinctum*. The presence of various *Fusarium* species in barley grain was also confirmed in earlier studies by Hudec and Roháčik [14], who reported the occurrence of *F. acuminatum*, *F. arthrosporioides*, *F. avenaceum*, *F. culmorum*, *F. chlamydosporum*, *F. equiseti*, *F. graminearum*, *F. langsethiae*, *F. nivale* (syn. *Microdochium nivale*), *F. oxysporum*, *F. poae*, *F. sambucinum*, *F. semitectum*, *F. solani*, *F. sporotrichioides*, and *F. tricinctum* in Slovakia, with *F. poae* being the most frequently identified species. Mycological analyses of barley grain conducted by Beccari et al. [15,16] enabled the determination of the species composition of both pathogenic and saprotrophic fungi, with a predominance of *Alternaria* and *Fusarium* spp. These authors also identified isolates belonging to the genera *Aspergillus*, *Penicillium*, and *Epicoccum* in barley grain [15]. In turn, Romero-Cortes et al. [17] isolated *Pyrenophora teres* and *Cochliobolus sativus* from barley grain.

Fungi of the genus *Fusarium* are the primary source of mycotoxins in small-grain cereal crops cultivated in a temperate climate. These fungi produce metabolites such as deoxynivalenol (DON), zearalenone (ZEA), T-2 toxin, and HT-2 toxin [18]. Drakopoulos et al. [5] reported that certain agronomic practices may reduce the occurrence of pathogens responsible for FHB and lower the risk of *Fusarium* toxin contamination of barley grain, but they may simultaneously affect the accumulation of other fungal metabolites. They also observed that strobilurin fungicides contribute to increased concentrations of DON and beauvericin (BEA) in grain. According to Beccari et al. [19] and Shah et al. [20], geographical region and climatic conditions considerably affect the occurrence of FHB pathogens and, consequently, the diversity of mycotoxins present in grain. In the case of *F. graminearum*, the risk of FHB and contamination with the associated mycotoxins can be most effectively reduced through agronomic practices such as crop residue management combined with conventional tillage, appropriate crop rotation, and the selection of less susceptible cultivars [20]. Most *Fusarium* species spread through the dissemination of conidia (asexual spores), but *F. graminearum* has a potential epidemiological advantage because it is also capable of forming perithecia containing ascospores that persist on crop residues (teleomorph: *Gibberella zeae*) [21]. Fungicide protection is an effective strategy for controlling FHB; however, the efficacy of individual fungicides varies and depends on multiple factors during application, including the uniformity of plant growth stage (such as flowering), weather conditions during treatment, and the efficacy of the active ingredient against a specific fungal isolate [22]. The protective effects of the fungicide chlorothalonil and the systemic fungicides prothioconazole and pyraclostrobin, applied individually or in combination, were quantitatively assessed in a field study conducted in the United Kingdom [23]. According to the cited authors, in the absence of fungicide treatments, the severity of FHB symptoms in spring barley was minimized by growing cultivars with good disease resistance. They also found that yield increases in fungicide-protected treatments comprised two distinct components. The first was an increase in the number of grains per 1 m^2^ (4.3–7.5%), which was elicited by the application of prothioconazole and pyraclostrobin, but not chlorothalonil. The second component was an increase in mean grain weight (3.7–4.6%) following the application of each of the tested fungicides. Fernandez et al. [24] noted that a comprehensive strategy for preventing the spread of *F. graminearum*, the main causative agent of FHB, can be developed by analyzing the efficacy of fungicides against this pathogen. In turn, Matengu et al. [25] emphasized that FHB can be controlled with fungicides at the flowering stage to reduce disease symptoms and mycotoxin accumulation, thereby lowering yield losses. However, the use of fungicides may be economically and environmentally unjustified when weather conditions are not conducive to FHB development. The cited authors concluded that fungicides should be used only when the pathogen is present and when weather conditions increase the risk of infection. They also highlighted that the risk of FHB should be modeled based on weather data to facilitate integrated crop disease management in production systems, and, above all, to maximize the efficacy of fungicides, while reducing their environmental impacts.

In view of the above, the aim of this study was to determine the spectrum of microscopic fungi and the presence of mycotoxins in the grain of fungicide-treated spring barley grown in northeastern Poland.

## 2. Results

### 2.1. Weather Conditions During the Study

**Figure 1:**
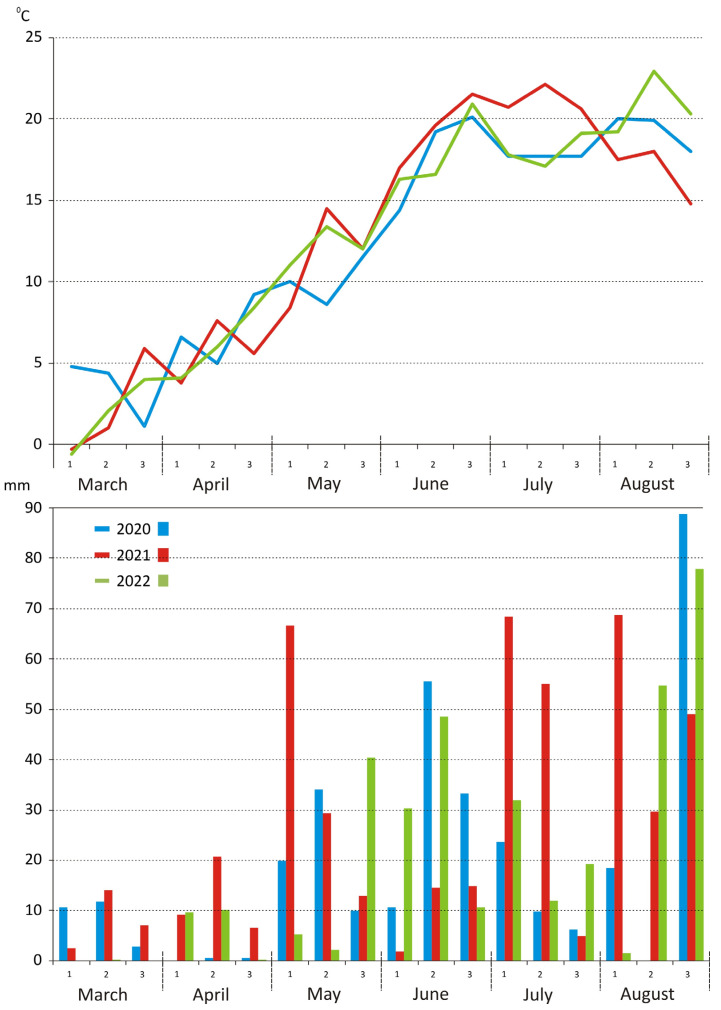
Precipitation and mean weekly temperatures during growing seasons in successive years of the experiment.

Mean daily temperatures during the growing season (March to August) differed noticeably between the years. Mean temperatures during this period were similar in 2020 and 2022 (12.5 and 12.8 °C, respectively), but the mean temperature in 2021 was higher (14.9 °C) due to elevated temperatures in June and July (19.4 and 21.2 °C, respectively) (Figure 1). Total precipitation in successive growing seasons reached 355 mm in 2020, 476.1 mm in 2021, and 336.7 in 2022. In 2020, April was extremely dry. Rainfall distribution was uneven in all three years of the study. The greatest variations were noted in 2021. This year was characterized by the highest temperatures in June and July, which were accompanied by very high precipitation in July and the first ten days of August, which promoted the development of fungi colonizing grain.

### 2.2. Yield

Grain yields were similar in both cultivars across the study years. Average grain yield was significantly highest in 2020 (5.8 t/ha), and it did not differ significantly between 2021 and 2022 (4.2 and 4.5 t/ha, respectively) (Table 1). Grain yield was higher in fungicide-protected treatments. The fungicide-induced increase in grain yield reached approximately 10% in cv. Atrika and approximately 20% in cv. Vermont on average, and the latter difference was statistically significant. In cv. Atrika, no significant differences in grain yield were observed between the control and fungicide-protected treatments in successive years of the experiment. In contrast, in cv. Vermont, grain yield in 2020 and 2021 was approximately 24% and 35% higher in fungicide-protected treatments than in the control, respectively, and these differences were statistically significant (Table 1).

### 2.3. Isolation of Fungi

**Figure 2:**
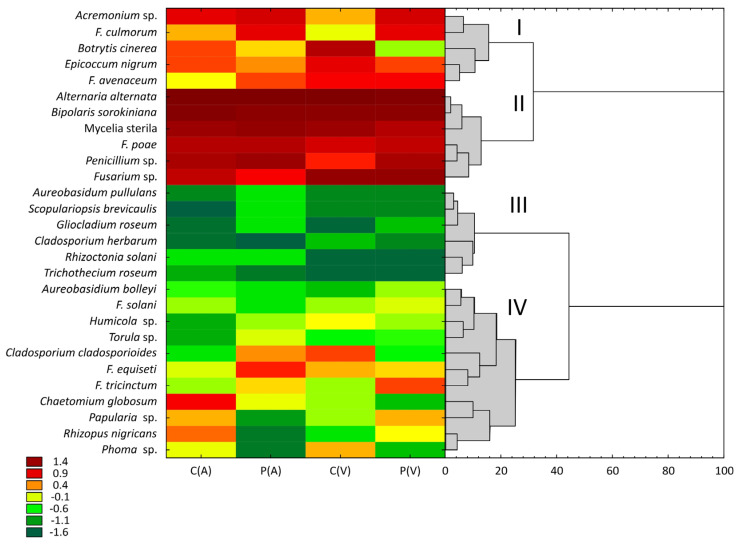
Hierarchical analysis of the abundance of fungal species/genera isolated from the grain of two spring barley cultivars harvested from control and fungicide-protected plants. C(A)–cv. Atrika, control; P(A)–cv. Atrika, fungicide protection; C(V)–cv. Vermont, control; P(V)–cv. Vermont, fungicide protection. Four distinct clusters (I–IV) were identified.

The grain mycobiome of both spring barley cultivars is presented in Table 2. Twenty-eight fungal species/genera (including *Mycelia sterilia*) were isolated in each year of the experiment (Table 2A). *Alternaria alternata* and *Bipolaris sorokiniana*, fungal species that commonly colonize cereal grain, were most abundant, with counts ranging from 132 to 471 and from 54 to 112 isolates, respectively. Their proportions ranged from 27% in 2020 to 45% in 2021 for *A. alternata*, and from 10% in 2022 to 18% in 2021 for *B. sorokiniana* (Table 2). Fungi of the genus *Fusarium* were also widely represented, accounting for 7% to 27% of all isolates, depending on the year. Interestingly, a clear increase in the abundance of these isolates was observed in successive years of the study, both in grain from control plants and from fungicide-protected plants (Table 2A,B; Figure 2). *Fusarium avenaceum*, *F. equiseti*, *F. culmorum*, *F. poae*, *F. tricinctum*, and *F. solani* were isolated in each year. In contrast, isolates of *F. graminearum*, *F. sporotrichioides*, *F. oxysporum*, *F. dimerum*, and *Microdochium nivale* (= *F. nivale*) were detected sporadically, and their impact on the concentrations and composition of mycotoxins in grain was negligible. Isolates that were unambiguously assigned to the genus *Fusarium*, but were not identified to species level, accounted for a substantial proportion of all isolates (ranging from 24% in 2022 to 45% in 2021).

### 2.4. Mycotoxin Content of Grain

A total of 45 metabolites produced mainly by *Fusarium* spp. and *Alternaria* spp. were identified in barley grain in each year of the three-year experiment (Table 3 and Table S1). The identified mycotoxins were classified into four groups. The first group comprised 11 major *Fusarium* toxins, including group B (DON, D3G, NIV, NIV3G) and group A trichothecenes (T-2, HT-2, HT-2G, MAS, DAS, NEO), as well as moniliformin (MON). The second group consisted of 20 *Fusarium* toxins belonging to different classes of chemical compounds (BEA, BEA-A, EN A, EN A1, EN B, EN B1, EN B2, CUL, 15-HCUL, ABY, API, AFU, CHIOL, CHOL, CHRY, EQU, FUN, SAM, SIC, and W493). The third group comprised six *Alternaria* toxins (TEA, AOH, AME, TEN, ALT, and INF), and the fourth group contained eight other metabolites (ZDIOL, ZNIOL, BI-A, ABA, Ili-H, MCER, EMO, and TRY).

The concentrations of fungal metabolites identified in grain from all treatments in each year of the study are presented in Table 1, whereas the mean concentrations of these metabolites for the entire three-year experiment are shown in Table 3. Fungicide protection did not lead to a significant reduction in the concentrations of toxins belonging to the first group, and even increased their levels by around 10%. Fungicides decreased the concentrations of NIV3G (by more than three-fold), NIV (by 32.6%), and DON (by 26.4%). The concentrations of group A trichothecenes and MON increased under fungicide protection. Marked but statistically non-significant differences were observed between the examined cultivars. The concentrations of the major *Fusarium* toxins were substantially higher in the grain of cv. Vermont, originating from both control and fungicide-treated plants (by 148% and 142%, respectively), than in the grain of cv. Atrika. However, the absolute concentrations of these metabolites were low. Greater differences in the content of other *Fusarium* toxins (group II) were observed between barley cultivars. In grain harvested from both control and fungicide-treated plants, the total concentrations of these metabolites were significantly higher in cv. Vermont (Table 3), and their accumulation was slightly lower in control grain than in the grain harvested from fungicide-protected plants. The concentrations of toxins produced by fungi of the genus *Alternaria* (group III) and the remaining identified metabolites (group IV) did not differ significantly between barley cultivars or between control and fungicide-protected treatments (Table 3).

Non-parametric Friedman ANOVA revealed significant differences in the total concentrations of eight toxins (Table S1). The grain sample of cv. Atrika harvested from fungicide-protected plants was destroyed in 2020; therefore, the total values for that year were respectively lower. Despite the above, the concentrations of the major toxins (group I) were lowest in grain harvested in 2022. Moniliformin was the dominant toxin in group I, and its concentrations in the grain of cv. Vermont from fungicide-protected treatments ranged from 95.27 μg kg^−1^ in 2022 to 596 μg kg^−1^ in 2021. The predominant group II toxins were cyclodepsipeptide metabolites, in particular enniatin B (ENN B) and enniatin B1 (ENN B1). In the grain of cv. Vermont harvested from fungicide-protected treatments, the concentrations of these metabolites ranged from 238.16 in 2022 to 687.12 μg kg^−1^ in 2020 (ENN B) and from 84.08 in 2022 to 600.08 μg kg^−1^ in 2020 (ENN B1). In the group of Alternaria toxins (group III), INF was the predominant metabolite, and its concentrations in the grain of cv. Vermont ranged from 115.77 μg/kg (control plants in 2020) to 338.34 μg/kg (fungicide-protected plants in 2022).

The significance of differences in the concentrations of the analyzed metabolites across the experimental years was assessed using Friedman ANOVA because the data were not normally distributed and parametric tests could not be applied. Friedman ANOVA revealed significant differences in the concentrations of eight metabolites (NIV, HT-2G, ENA, ENB, SIC, W493, INF, and ZDIOL) across years (Table S1).

**Figure 3:**
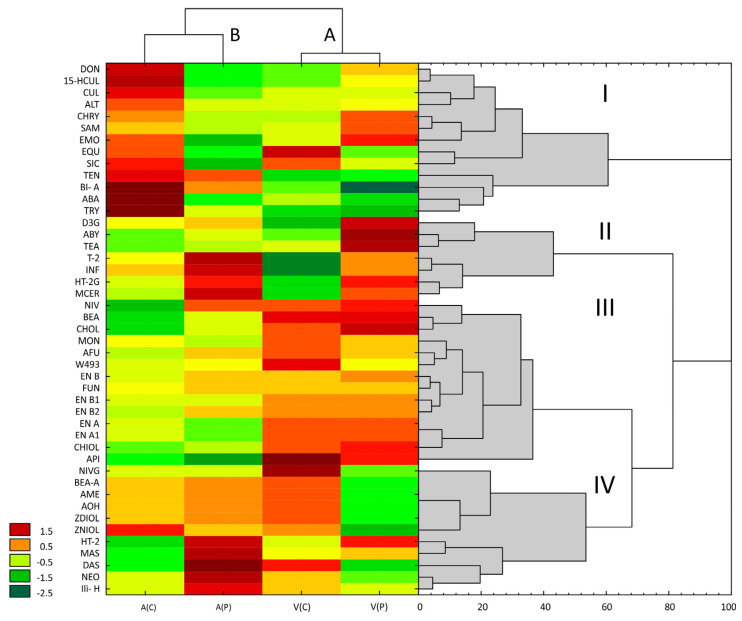
Hierarchical analysis of the concentrations of metabolites identified in the grain of two spring barley cultivars harvested from control and fungicide-protected plants. C(A)–cv. Atrika, control; P(A)–cv. Atrika, fungicide protection; C(V)–cv. Vermont, control; P(V)–cv. Vermont, fungicide protection. Metabolite abbreviations are given in the Abbreviations section. Four distinct clusters (I–IV) for mycotoxins and two (A,B) for cultivars were identified.

Mycotoxin concentrations in the grain of two barley cultivars were subjected to a hierarchical analysis (Figure 3), which enabled the identification of four distinct clusters. Interestingly, the identified toxins were not discriminated by their chemical structure or the corresponding fungal species. Cluster III contained nearly all cyclodepsipeptides (excluding BEA A); cluster IV comprised most group A trichothecenes (except for T-2 and HT-2G), whereas cluster I was characterized by particularly high concentrations of, among others, group B trichothecenes (DON, CUL, and 15HCUL), *Fusarium* toxins (SAM, CHRY, SIC, EQU, and SAM), and *Alternaria* toxins (ALT, TEN, EMO, ABA, and TRY). Cluster II was the smallest group, containing, among others, D3G, T-2, HT-2G, AbY, and *Alternaria* toxins TEA and INF.

Hierarchical clustering performed along the second dimension produced two distinct clusters corresponding to the studied cultivars. However, grain samples did not cluster according to fungicide treatment (control vs. protected) (Figure 3). Cluster A comprised both treatment variants for cv. Vermont, and cluster B included both variants for cv. Artika. In almost all cases mycotoxin concentrations did not exceed the permissible limits in any of the analyzed grain samples.

## 3. Discussion

Barley is an important cereal crop cultivated worldwide for food, feed, malting, and brewing. Grain contamination by fungi and mycotoxins remains a major concern because it directly affects grain quality, safety, and suitability for processing [5,26]. The prevalence of fungal pathogens is strongly influenced by environmental conditions during the growing season, including temperature and rainfall distribution [20,27]. In the present study, uneven rainfall and high temperatures in June and July of 2021, combined with very high precipitation in late July and early August, likely promoted the development of fungal pathogens on barley grain. Similar observations have been reported in other studies, demonstrating that temperature and moisture during flowering and grain filling are critical for the development of pathogens belonging to the *Fusarium* head blight (FHB) complex [15,28]. Weather conditions also had a significant impact on grain yield. The highest yield was observed in 2020 (5.8 t/ha), whereas yields in the other years did not differ significantly. This is consistent with previous reports from northeastern Poland, where precipitation patterns during key developmental stages strongly influenced spring barley yields [29,30]. Reproductive phases such as spike formation and flowering are particularly sensitive to environmental stresses, accounting for a large portion of yield variability [30].

Fungicide application generally increased grain yield compared with untreated control plots. This effect aligns with previous findings showing yield improvements following foliar fungicide applications [29,31,32]. However, fungicide efficacy depends on multiple interacting factors, including weather conditions, infection severity, cultivar susceptibility, and application timing [33]. In barley, effective control of FHB requires attention to flowering biology and exposure of floral tissues to pathogen spores [33,34]. Combined application of multiple fungicides during the growing season is often recommended to enhance efficacy and limit the development of pathogen resistance [35,36,37]. The fungal community associated with barley grain in this study was dominated by *Alternaria alternata* and *Bipolaris sorokiniana*, with additional presence of *Fusarium* spp. While species-level identification of *Fusarium* is challenging and may require molecular approaches, the genus-level assessment used here is widely accepted in ecological and phytopathological surveys and allows reliable evaluation of pathogen occurrence [16,38]. The proportion of *Fusarium* spp. increased over the three-year period, consistent with other studies reporting that environmental conditions and cultivation practices influence the abundance of these pathogens [27,38]. A broad spectrum of fungal metabolites was detected in barley grain. In total, 45 metabolites produced mainly by *Fusarium* and *Alternaria* spp. were identified, with nivalenol (NIV), enniatins (ENN B and ENN B1), HT-2 glucoside, and moniliformin among the most abundant. These results agree with previous reports on barley grain, where enniatins, NIV, and HT-2/T-2 toxins were frequently detected [16,39,40,41]. The occurrence and concentration of mycotoxins were strongly influenced by environmental conditions, particularly temperature and humidity during flowering and grain maturation [42,43,44]. Fungicide application did not consistently reduce mycotoxin levels. In some cases, slightly higher concentrations of specific toxins were observed in treated plots, in line with prior observations that reductions in visible disease symptoms do not always correspond to lower mycotoxin contamination [35,45]. This may reflect stress responses in both plants and fungi or suboptimal timing of fungicide applications. Overall, weather conditions appeared to exert a stronger influence on mycotoxin accumulation than fungicide treatment alone. Taken together, these results indicate that environmental conditions were the primary factor determining fungal colonization and mycotoxin contamination of barley grain. Uneven rainfall distribution and high temperatures during sensitive growth stages favored fungal development, while fungicide application improved grain yield but had limited or variable effects on mycotoxin concentrations. These findings highlight the importance of optimizing crop protection strategies by considering cultivar-specific flowering patterns, appropriate timing, and potential combinations of fungicide active ingredients. This data also reinforces the need for ongoing monitoring of fungal communities and mycotoxin profiles to support safe and high-quality barley production.

Fungicide protection did not reduce group I toxin concentrations and was even associated with a minor increase (~10%). Caldwell et al. [38] reported that reduced FHB symptoms after fungicide application did not always decrease DON in grain. Pyraclostrobin at ZGS 39 and a prothioconazole + tebuconazole mix at ZGS 60 were most effective in improving yield, quality, and reducing DON. Karron et al. [27] found low DON, HT-2, and T-2 levels in barley, though DON appeared in all treatments in 2012–2013 and only in control and tebuconazole-treated grain in 2014; HT-2 and T-2 were absent in grains treated with multi-active fungicides. Gozzi et al. [46] demonstrated that DON and ENN B were the most frequently detected mycotoxins in 2020–2021. Perkowski et al. [47] analyzed six cereal species (common wheat, durum wheat, triticale, rye, oats, and barley) and also reported the lowest total mycotoxin concentration (25.56 mg/kg) in barley grain, particularly when compared with durum wheat (151.89 mg/kg). The mean concentrations of the most abundant mycotoxins were 37 mg/kg for DON, 18 mg/kg for 3-AcDON, and 15 mg/kg for 15-AcDON [47]. Gil-Serna et al. [48] reported the presence of DON and ZEN in 72% and 38% of barley grain samples, respectively, at concentrations below the maximum levels permitted in the EU, whereas NIV and HT-2/T-2 were identified in 17% and 10% of samples, respectively. In the present study, fungicide protection exerted contrasting effects. Fungicides induced more than a threefold reduction in NIV3G levels, and reduced NIV and DON concentrations by 32.6% and 26.4%, respectively. At the same time, the applied fungicides increased the concentrations of group A trichothecenes and MON. Differences between the studied cultivars were notable but not statistically significant, and the overall toxin concentrations remained low. More pronounced differences between cultivars were observed for other *Fusarium* toxins (group II). In both fungicide-treated and control plants, the total concentrations of these metabolites were significantly higher in cv. Vermont, while control grain accumulated slightly lower amounts than grain from fungicide-protected treatments. In a two-year study by Habschied et al. [45], DON, CUL, 15-HCUL, 5-HCUL, and AFU were not detected in brewing barley grain in the dry year of 2020, whereas two mycotoxins (SIC and INF) and the non-specific metabolite tryptophol were identified in both years, regardless of the applied protective treatment. Interestingly, control grain was characterized by lower DON concentrations that grain harvested from fungicide-protected treatments. In cv. Bravo, this was the case for all the mycotoxins whose concentrations were lower in the control sample than in treated samples. DON levels were significantly higher after the combined application of prothioconazole and tebuconazole (1809 μg/kg) relative to the control sample (196 μg/kg), reaching almost 90% in cv. Favorit. Similar trends were observed for CUL, 15-HCUL, and 5-HCUL. According to the cited authors, antifungal treatments do not effectively inhibit the synthesis of INF and SIC, even in dry years, and both toxins commonly occur in barley grain, particularly in humid years [45]. The concentrations of the remaining identified mycotoxins, including *Alternaria* toxins, did not differ significantly between cultivars or between control and fungicide-protected treatments.

Spring barley cultivated in a field experiment in northeastern Poland achieved high grain yields, and fungicide protection contributed to increased yields. These results can also be attributed to weather conditions. Regardless of fungicide application, grain was predominantly colonized by *A. alternata*, *B. sorokiniana*, and *Fusarium* spp. Fungicide protection did not significantly reduce mycotoxin concentrations, and a slight increase of around 10% was even observed in the levels of the major *Fusarium* toxins (group I). Weather conditions exerted a greater impact on mycotoxin levels, particularly uneven rainfall distribution across the study years, and high temperatures in June and July of 2021, which were accompanied by very high precipitation in July and the first ten days of August.

## 4. Conclusions

This three-year study with two spring barley cultivars demonstrated that fungicide treatment significantly improved grain yield, with a greater response observed in cv. Vermont than in cv. Atrika. Despite this agronomic benefit, fungicide application did not lead to a reduction in the overall contamination of grain by toxigenic fungi or total *Fusarium* mycotoxin concentrations. It is a kind of ‘fungicide paradox’. Although fungicide use reduced the concentrations of selected toxins such as nivalenol, nivalenol-3-glucoside, and deoxynivalenol, it was associated with an increase in group A trichothecenes and moniliformin. Moreover, fungicide treatment did not decrease the total *Fusarium* toxin load and in some cases slightly increased it. Additionally, significant year-to-year variation in eight mycotoxins highlights the strong influence of environmental conditions on fungal colonization and toxin production. The mycological analysis revealed that *Alternaria alternata* and *Bipolaris sorokiniana* were the predominant fungal species colonizing the grain, while *Fusarium* spp. were consistently present and constituted a substantial proportion of isolates. Chemical protection modified the mycotoxin profile rather than eliminating contamination.

## 5. Material and Methods

This study was conducted on two spring barley cultivars, Artika (KWS, Poznań, Poland) and Vermont (KWS, Poznań Poland), grown for feed purposes. Both cultivars are characterized by high yield potential and moderate resistance to fungal pathogens that cause powdery mildew, rusts, and net blotch. The cultivar Artika exhibits low resistance to net blotch and scald.

### 5.1. Field Experiment

A three-year field experiment was conducted in 2020–2022 at the Agricultural Experiment Station in Bałcyny (Bałcyny, DMS: 53° 35′49″ N 19° 51′15″ E). The soil was suitable for barley cultivation, and the preceding plant was winter raps. Meteorological data (mean daily temperature and total precipitation) were acquired with the use of the PM Ecology automatic weather station (PM Ecology Ltd., Gdynia, Poland) in the Agricultural Experiment Station (AES) in Bałcyny, where a three-year field experiment was conducted.

The experiment had a randomized complete block design with four replications. Plot area was 20 m^2^. Each year, spring barley was sown in the last ten days of March at a rate corresponding to a plant density of 350 plants per m^2^. N/P/K fertilizers were applied at 80/30/83 kg/ha, with the N rate split into 50 kg/ha applied pre-sowing and 30 kg/ha applied as top dressing in growth stage BBCH 31 [49]. Weeds were managed with the herbicide Mustang 306 SE (Corteva/DowAgro, Warsaw, Poland) according to the manufacturer’s recommendations for spring barley. The following fungicides were applied: Capalo 337.5 SE (BASF Sp. z o.o, Warsaw, Poland) (fenpropimorph—200 g/L, epoxiconazole—62.5 g/L, metrafenone—75 g/L; BASF, Poland) in the stem elongation stage (BBCH 33–37) and Adexar Plus (BASF Sp. z o.o, Warsaw, Poland) (fluxapyroxad—41.6 g/L, epoxiconazole—41.6 g/L, pyraclostrobin—66.6 g/L; BASF, Poland) in the middle of heading (BBCH 55). In response to cereal leaf beetle infestation, the crops were sprayed with the insecticide Decis Mega (deltamethrin 50 g/L; Bayer CropScience, Warsaw, Poland) in mid-June. Spring barley was harvested in the fully ripe stage using a Wintersteiger Classic (Ried im Innkreis, Austria) plot combine harvester.

### 5.2. Isolation and Identification of Fungi Colonizing Grain

Each year, 800 kernels were randomly selected from each treatment for phytopathological analysis (a total of 3200 kernels per year). The grain was thoroughly rinsed with distilled water and disinfected with EtOH (70%, 5 min) and NaOCl (1%, 5 min), after which the samples were rinsed three times with sterile distilled water. The prepared kernels were then plated onto sterile Petri dishes (94 × 16 mm, Merck, Poland) containing PDA medium (Merck, Warsaw, Poland), at ten kernels per plate. Incubation was carried out at a temperature of 20–23 °C for 7–10 days. Small disks (of 5 mm in diameter) overgrown with mycelium, cut out from seven-day-old PDA cultures of fungi, were placed in the center of each Petri dish containing the same medium. After approximately 14–20 days of growth (depending on the isolate, sometimes only after 30 days), the resulting fungal cultures were identified to genus and species level under a light microscope (Labophot 2A, Nikon, Tokyo, Japan) based on their morphological characteristics, using monographic keys [50,51,52].

### 5.3. Mycotoxin Analysis by LC-MS/MS

The mycotoxin analysis was performed according to the method described by Suchowilska et al. [10]. *Fusarium* mycotoxins were extracted in a rotary shaker with a dilution solvent composed of acetonitrile/water/acetic acid (79:20:1 *v*/*v*/*v*), applied at 20 mL per 5 g of grain for 90 min. The extracts were transferred to glass vials using Pasteur pipettes, and 350 μL aliquots were diluted with the same volume of the dilution solvent (acetonitrile/water/acetic acid, 20:79:1, *v*/*v*/*v*).

The extracts were stirred, and 5 μL of the diluted extract was injected into the LC–MS/MS system without further pre-treatment. For validation purposes, the entire procedure was simplified to decrease the amounts of standards required for spiking. *Fusarium* mycotoxins were identified and quantified according to the procedure described by Sulyok et al. [53] with the use of a QTrap5500 LC-MS/MS System (Applied Biosystems, Foster City, CA, USA) equipped with a TurboIon spray electrospray ionization (ESI) source and a 1290 Series UHPLC System (Agilent Technologies, Waldbronn, Germany). The analytes were separated on a Gemini C18 column (150 × 4.6 mm i.d., 5 μm particle size) with a 4 × 3 mm precolumn with the same characteristics (Phenomenex, Torrance, CA, USA). The analysis was performed using a fully validated method for identifying >500 mycotoxins and other secondary metabolites, as described by Sulyok et al. [53].

### 5.4. Statistical Analysis

Differences in the yield performance of the analyzed spring barley cultivars were assessed by ANOVA, and the significance of differences between means was evaluated using the SNK test. Data on grain colonization by microscopic fungi and mycotoxin concentrations were subjected to a multivariate hierarchical analysis, and the results were visualized as dendrograms and heatmaps. The significance of differences in the concentrations of the analyzed metabolites was assessed using non-parametric Friedman ANOVA, and pairwise comparisons were performed using the non-parametric Mann–Whitney U test. All statistical analyses were conducted in Statistica v. 13.3 (TIBCO Software Inc.) [54].

## Figures and Tables

**Table 1:** Grain yield (t/ha) of two spring barley cultivars in subsequent years of the experiment.

Year	Artika		Vermont		Mean
	C	P	C	P	
2020	5.29	5.90	5.32 ^b^	6.51 ^a^	5.8 ^A^
2021	4.54	5.29	2.71 ^b^	4.45 ^a^	4.2 ^B^
2022	4.09	4.28	4.71	4.93	4.5 ^B^
mean	4.64	5.16	4.25 ^Y^	5.30 ^X^	

C—non-treated control (without fungicides); P—fungicide protection (first treatment: Capalo 337.5 SE (BBCH 31-32); second treatment: Adexar Plus (BBCH 38-39). Means followed by different letters differ significantly at *p* < 0.05: lowercase letters (a, b) denote differences between treatments in cv. Vermont; superscripts (X, Y) denote differences between treatment means for cv. Vermont across years; capital letters (A, B) denote differences between years.

**Table 2:** (**A**) Fungi isolated from the grain of two spring barley cultivars in each year of the experiment. (**B**) Fungi isolated occasionally from the grain of two spring barley cultivars during the experiment.

(**A**)																
	**Year**	**2020**				**2021**						**2022**				
**No.**	**Cultivar**	**Artika**		**Vermont**		**Total**	**Artika**		**Vermont**		**Total**	**Artika**		**Vermont**		**Total**
	**Species/Genus**	**C**	**P**	**C**	**P**		**C**	**P**	**C**	**P**		**C**	**P**	**C**	**P**	
1	*Acremonium* sp.	12	12	3	13	40	8	7	6	4	25	4	1	1	2	8
2	*Alternaria alternata*	32	23	41	36	132	92	92	91	97	372	115	124	113	119	471
3	*Aureobasidium pullulans*		1	1	1	3	2	1			3		1	1		2
4	*Aureobasidium bolleyi*	2	1	2	2	7	1	1		3	5	2	1	1		4
5	*Bipolaris sorokiniana*	11	24	17	14	66	29	29	54	36	148	44	18	15	35	112
6	*Botrytis cinerea*	4	3	15	4	26	4	4	4		12	7	2	9	1	19
7	*Chaetomiun globosum*	16	4	3	1	24	3	1	3	1	8	3	3			6
8	*Cladosporium cladosporioides*	1	1	3	1	6	1	7	3	2	13	2	4	6		12
9	*Cladosporium herbarum*				1	1	1				1			3		3
10	*Epicoccum nigrum*			1		1	2			2	4	13	12	22	13	60
**11**	***Fusarium avenaceum***				**3**	**3**	**1**	**6**	**3**	**1**	**11**	**9**	**7**	**13**	**13**	**42**
**12**	***F. equiseti***		**1**	**2**	**6**	**9**	**1**	**7**	**3**	**2**	**13**	**7**	**7**	**5**	**3**	**22**
**13**	***F. culmorum***			**1**		**1**	**1**	**7**		**4**	**12**	**10**	**12**	**6**	**14**	**42**
**14**	***F. poae***			**2**		**2**		**3**	**8**	**6**	**17**	**26**	**18**	**14**	**21**	**79**
**15**	***F. solani***	**3**	**2**	**4**	**3**	**12**	**2**	**1**			**3**	**1**		**2**	**3**	**6**
**16**	***F. tricinctum***			**1**		**1**		**2**		**2**	**4**	**6**	**7**	**5**	**13**	**31**
**17**	***Fusarium*** **spp.**		**1**	**3**	**7**	**11**	**8**	**3**	**16**	**17**	**44**	**17**	**13**	**22**	**23**	**75**
18	*Gliocladium roseum*	1		1	1	3		2		1	3		1			1
19	*Humicola* spp.	2	1		2	5	1		4	2	7		4	4	1	9
20	*Papularia* sp.	4			2	6	2	1	4	2	9	5	1	2	9	17
21	*Penicillium* spp.	12	17	2	28	59	7	11	2	4	24	8	5	9	5	27
22	*Phoma* sp.	2		3	2	7	4	1	6		11	3		1		4
23	*Rhizoctonia solani*		1	1		2	2				2	2	2			4
24	*Rhizopus nigricans*	3	1		2	6			1		1	10		3	7	20
25	*Scopulariopsis brevicaulis*			1		1		2	1		3		1		1	2
26	*Torula* sp.	1	1	1	1	4	2	2	3	2	9		3	1	1	5
27	*Trichothecium roseum*		1			1	3				3			1		1
28	Mycelia sterilia	13	11	12	11	47	13	24	15	16	68	6	10	7	5	28
(**B**)																
	**Year**	**2020**					**2021**			**2022**						
**No.**	**Cultivar**	**Artika**		**Vermont**		**Total**	**Artika**		**Vermont**		**Total**	**Artika**		**Vermont**		**Total**
	**Species/Genus**	**C**	**P**	**C**	**P**		**C**	**P**	**C**	**P**		**C**	**P**	**C**	**P**	
1	*Alternaria tenuissima*	3				3						1				1
2	*Aspergillus niger*	4		6	3	13						1				1
3	*Chalara* sp.	*2*	2	2		6		3			3					
4	*Drechslera teres*											3	11	7		21
**5**	***F. dimerum***														**3**	**3**
**6**	***F. graminearum***				**1**	**1**						**1**	**1**		**2**	**4**
**7**	***F. nivale***											**1**				**1**
**8**	***F. oxysporum***							**3**			**3**				**2**	**2**
**9**	***F. sporotrichioides***								**1**		**1**	**4**			**1**	**5**
10	*Gelasinospora sphaerospermum*							1	4	2	7		1			1
11	*Gliocladium catenulatum*			1		1										
12	*Mucor* spp.	3				3								3		3
13	*Periconia macrospinosa*								1		1					
14	*Phytium* sp.						2				2					
15	*Sclerotinia sclerotiorum*			1		1								1		1
16	*Stachybotrys chatrarum*	17				17										
17	*Verticilium albo-atrum*	*1*	2			3										

C-non-treated control, P-fungicide protection (see section Material and Methods). *Fusarium* isolates are marked in bold.

**Table 3:** Average concentrations of metabolites (μg/kg) identified in the grain of two spring barley cultivars harvested from control (non-treated) and fungicide-protected plants in the years of the study (2020-2022) and divided into four groups: I-major *Fusarium* toxins; II-other *Fusarium* toxins; III-*Alternaria* toxins; and IV-other toxins and metabolites. If the concentration of the metabolite was below the LOD (see Table S1) then value of 0.5·LOD was used for the calculation of the mean.

Toxin Group	Toxin	Control			Protection		
		Artika	Vermont	Mean	Artika	Vermont	Mean
I	DON	24.23	13.14	18.7	0.65	24.17	14.8
	D3G	0.65	0.65	0.7	0.65	7.07	4.5
	NIV	10.85	128.23	69.5	28.75	68.13	52.4
	NIV3G	1.50	46.62	24.1	1.50	8.82	5.9
	T-2	1.59	1.88	1.7	4.26	2.25	3.1
	HT-2	4.77	10.79	7.8	10.88	12.78	12.0
	HT-2G	6.43	7.31	6.9	11.51	19.23	16.1
	MAS	1.03	7.09	4.1	6.49	7.33	7.0
	DAS	0.09	0.77	0.4	0.76	0.36	0.5
	NEO	0.28	0.43	0.4	0.45	0.32	0.4
	MON	157.40	299.90	228.7	145.08	376.46	283.9
II	BEA	0.67 b	3.92 a	2.3	0.65 b	1.49 a	1.2
	BEA-A	0.01	0.21	0.1	0.01	0.01	0.0
	EN A	1.97	6.80	4.4	1.48	7.12	4.9
	EN A1	27.69	61.12	44.4	24.36	83.60	59.9
	EN B	430.51	517.41	474.0	411.28	537.09	486.8
	EN B1	181.76	284.96	233.4	168.64	376.80	293.5
	EN B2	24.91	34.54	29.7	24.36	54.55	42.5
	CUL	25.06	23.62	24.3	8.21	23.99	17.7
	15-HCUL	64.13	26.12	45.1	4.30	32.75	21.4
	ABY	2.05	17.66	9.9	2.19	176.86	107.0
	API	2.15 b	8.99 a	5.6	0.25 b	7.43 a	4.6
	AFU	24.16	179.46	101.8	25.24	246.23	157.8
	CHIOL	0.01 b	2.31 a	1.2	0.01 b	4.95 a	3.0
	CHOL	0.60 b	18.50 a	9.5	6.13 b	42.02 a	27.7
	CHRY	104.80	128.22	116.5	94.72	179.81	145.8
	EQU	2.36	9.06	5.7	0.06	2.41	1.5
	FUN	0.04	0.28	0.2	0.04	0.37	0.2
	SAM	0.03	0.05	0.0	0.01	0.07	0.0
	SIC	190.75	297.47	244.1	15.00	161.88	103.1
	W493	3.09	6.17	4.6	2.93	5.75	4.6
III	TEA	1.05 b	115.92 a	58.5	1.05 b	20.88 a	12.9
	AOH	0.04	2.63	1.3	0.04	0.04	0.0
	AME	0.02	0.19	0.1	0.02	0.02	0.0
	TEN	1.51	0.82	1.2	1.28	0.95	1.1
	ALT	0.77	0.88	0.8	0.21	1.92	1.2
	INF	251.45	222.94	237.2	295.81	271.90	281.5
IV	ZDIOL	0.10	3.18	1.6	0.10	0.10	0.1
	ZNIOL	8.71	15.07	11.9	1.10	1.10	1.1
	BI- A	2.03	1.84	1.9	1.70	1.60	1.6
	ABA	30.08	24.81	27.4	20.57	25.41	23.5
	Ili- H	0.27	1.17	0.7	1.21	0.95	1.1
	MCER	0.59	0.80	0.7	0.19	0.19	0.19
	EMO	0.24	0.38	0.3	0.03	0.32	0.2
	TRY	26.40	18.79	22.6	16.68	18.38	17.7
Means	I	18.98	46.98	33.00	19.18	47.90	36.42
	II	54.34 A	81.34 B	67.84	39.49 B	97.26 A	74.16
	III	42.47	57.23	49.85	49.74	49.29	49.45
	IV	8.55	8.26	8.39 X	5.20	6.01	13.25 Y
	Σ	124.34	193.81	159.08	113.61	200.46	173.28

Means followed by different letters differ significantly at *p* < 0.05: lowercase letters (a, b) denote differences between cultivars within treatment; capital letters denote differences between cultivars (A, B) and treatments (X, Y) within specific toxin group. For toxin abbreviations, see Abbreviations section.

## Data Availability

The original contributions presented in this study are included in the article/Supplementary Materials. Further inquiries can be directed to the corresponding author(s).

## Supplementary Materials

The following supporting information can be downloaded at: https://www.mdpi.com/article/10.3390/toxins18040164/s1, Table S1: Concentrations of metabolites (mg kg^−1^) identified in the grain of two spring barley cultivars in the experiment along with the LOD and LOQ values. C-non-treated control, P-fungicide protection with (see: section Material and Methods). Statistically significant differentiation between years was confirmed by Friedman ANOVA for: D3G, HT-2G, EN A, EN B1, SIC, W493, INF and ZDIOL (relevant ranked values are highlighted in yellow in columns S, T and U). I…IV—toxin group.

## Abbreviations

The following abbreviations are used in this manuscript:

15-HCUL	15-hydroxyculmorin
5-HCUL	5-hydroxyculmorin
ABA	Abscisic acid
ABY	Antibiotic Y
AFU	Aurofusarin
ALT	Altenuene
AME	Alternariol monomethyl ether
AOH	Alternariol
API	Apicidin
BEA	Beauvericin
BEA-A	Beauvericin A
CHIOL	Chlamydospordiol
CHOL	Chlamydosporol
CHRY	Chrysogin
CUL	Culmorin
D3G	Deoxynivalenol 3-glucoside
DAS	Diacetoxyscirpenol
DON	Deoxynivalenol
EMO	Emodin
EN A	Enniatin A
EN A1	Enniatin A1
EN B	Enniatin B
EN B1	Enniatin B1
EN B2	Enniatin B2
EQU	Equisetin
FHB	Fusarium head blight
FUN	Fungerin
HT-2	HT-2 toxin
HT-2G	HT-2 toxin-2-glucoside
Ili-H	Ilicicolin H
INF	Infectopyron
LC-MS/MS	Liquid Chromatography with tandem mass spectrometry
MAS	Monoacetoxyscirpenol
MCER	Monocerin
MON	Moniliformin
NEO	Neosolaniol
NIV	Nivalenol
NIN-3G	Nivalenol 3-glucoside
SAM	Sambutoxin
SIC	Siccanol
T-2	T-2 toxin
TEA	Tenuazonic acid
TEN	Tentoxin
TRY	Tryptophol
W493	Cyclodepsipetide W493
ZDIOL	Zinndiol
ZEA	Zearalenone
ZNIOL	Zinniol
